# TATA boxes in gene transcription and poly (A) tails in mRNA stability: New perspective on the effects of berberine

**DOI:** 10.1038/srep18326

**Published:** 2015-12-16

**Authors:** Zhi-Yi Yuan, Xi Lu, Fan Lei, Yu-Shuang Chai, Yu-Gang Wang, Jing-Fei Jiang, Tian-Shi Feng, Xin-Pei Wang, Xuan Yu, Xiao-Jin Yan, Dong-Ming Xing, Li-Jun Du

**Affiliations:** 1 MOE Key Laboratory of Protein Sciences, Laboratory of Molecular Pharmacology and Pharmaceutical Sciences, School of Life Sciences and School of Medicine, Tsinghua University, Beijing 100084, China; 2 MD Anderson Cancer Center, University of Texas, Houston, Texas 77030, USA

## Abstract

Berberine (BBR) is a natural compound with variable pharmacological effects and a broad panel of target genes. We investigated berberine’s pharmacological activities from the perspective of its nucleotide-binding ability and discovered that BBR directly regulates gene expression by targeting TATA boxes in transcriptional regulatory regions as well as the poly adenine (poly (A)) tail at the mRNA terminus. BBR inhibits gene transcription by binding the TATA boxes in the transcriptional regulatory region, but it promotes higher levels of expression by targeting the poly (A) tails of mRNAs. The present study demonstrates that TATA boxes and poly (A) tails are the first and second primary targets by which BBR regulates gene expression. The final outcome of gene regulation by BBR depends on the structure of the individual gene. This is the first study to reveal that TATA boxes and poly (A) tails are direct targets for BBR in its regulation of gene expression. Our findings provide a novel explanation for the complex activities of a small molecule compound in a biological system and a novel horizon for small molecule-compound pharmacological studies.

As a natural isoquinoline molecule, berberine (BBR) is mainly applied in intestinal infections such as bacterial enteritis and dysentery^1^. Modern pharmacological studies have shown that BBR has diverse biological activities, including anti-hyperlipidemia^2^, anti-hyperglycemia^3^, anti-cerebral ischemia^4^, prevention and treatment of neurodegeneration in Alzheimer’s disease and Parkinson’s disease^5^,^6^, and even anti-tumor effects^7^. However, the diversity of BBR targets that mediate these pharmacological activities are not well understood.

In the 1960s, chemists discovered BBR’s ability to bind DNA with a thymidine-adenine preference *in vitro*^8^. We previously explored BBR’s effect on TATA boxes in a biological system^9^. DNA TATA box elements and mRNA poly (A) tails are pivotal in the regulation of gene expression^10^. The fact that BBR affects both mRNA and DNA in a living cell system is an important point that has been largely ignored in the exploration of BBR’s pharmacological activities. To explain BBR’s multiple functions, we conducted a comprehensive study based on the interaction between BBR and nucleotides both *in vivo* and *in vitro*. Our findings provide novel insight into the broad pharmacological effects of BBR.

## Results and discussion

### Binding affinities on the nucleotides

**Figure 1:**
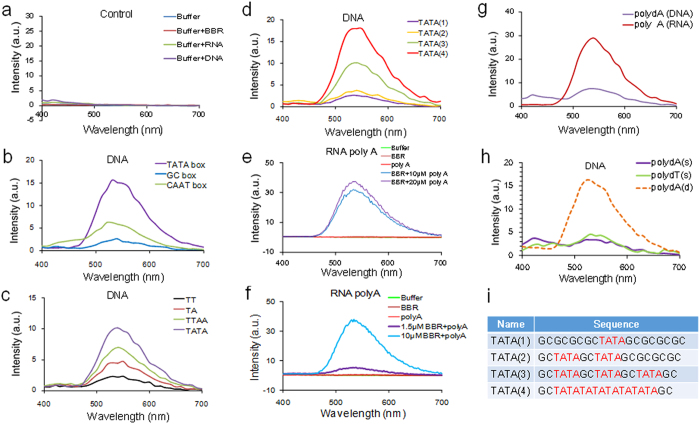
Characteristics of BBR’s affinities for DNA and RNA sequences. (**a**): Controls for buffer, BBR, RNA and DNA. (**b**): TATA box, GC box and CAAT box. (**c**): Different TA compositions. (**d**): Different TATA boxes. In (**a–d**), the DNA oligos were applied at the same concentration (25 μM). (**e**): Poly (A). (**f**): Different concentration of BBR with poly (A) (20 μM). (**g**): Comparison of poly (A) of deoxyadenine (poly dA) (10 μM) and poly (A) of adenine (poly A) (10 μM). (**h**): Comparison of poly dA as a single strand (10 μM, s-30 nt) and a double strand (5 μM, d-30 bp). In (**a–h**), BBR was applied at the same concentration (10 μM). (**i**): Sequences containing TATA boxes used in (**d**).

Due to BBR’s reported thymidine-adenine preference when binding to nucleotides, we first screened the binding affinities of BBR to *in vitro*-synthesized *cis*-transcriptional elements with different “TA” percentages. The binding affinity sequence, from strong to weak, was TATA box > CAAT box > GC box (Fig. 1a,b). The ability of BBR to bind TT, TA, TTAA and TATA sequences increased with increasing repetitions of the target sequences (Fig. 1c). Consistent with the “TATA” preference, BBR’s TATA binding affinity was positively correlated with the number of TATA repeats (Fig. 1d,i; Supplementary Table S1), indicating that different compositions of the “TA” variant might account for the differing effects of BBR on individual genes *in vivo*.

BBR also binds to RNA *in vitro*, with a preference for adenosine (A)/uridine (U)^11^. BBR physically interacts with the poly (A) tail of mRNA in a concentration-dependent manner (Fig. 1e,f). BBR showed stronger binding affinity to single strand poly-adenosine than a single strand poly-deoxyadenosine (dA) (Fig. 1g; Supplementary Table S2). Nuclear magnetic resonance (NMR) spectroscopy analyses (a 2D NMR for ^1^H-^13^C cosy (HMBC)) revealed that BBR had a stronger binding affinity for single-stranded poly-adenosine, as several chemical shift changes emerged when AMP^2-^ (monomer of poly (A)) was mixed with BBR (Supplementary Fig. S1). However, BBR had increased affinity for a complementary double strand of deoxyadenosine with deoxythymidine (dT) (Fig. 1h), indicating that BBR may bind to double-stranded DNA via a different mechanism, which might depend on the intercalation of BBR into the minor groove of the DNA double strand^8^. We might confirm that BBR showed the preference to a complementary double strand of deoxyadenosine with deoxythymidine and to a single stand of adenosine (poly (A)) *in vitro*, thus providing the targeting basis for BBR.

### BBR effects on “TATA” box and poly (A) tail in wild type cells

**Figure 2:**
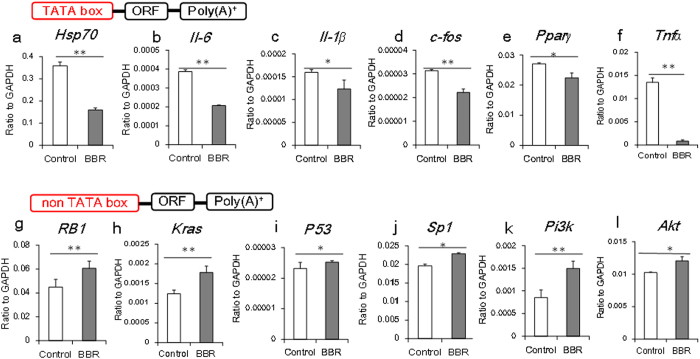
mRNA expression of cytokines with or without a TATA box. PC12 cells were treated with BBR for 15 h, and the expression of several mRNAs was detected by qPCR. (**a–f**): mRNA expression of genes containing a TATA box. (**g–l**): mRNA expression of genes without a TATA box. The data are expressed as the mean ± S.D. from six independent experiments. *, **P < 0.05, *P* < 0.01 *vs.* control groups.

According to the central dogma, both DNA and mRNA are important genetic materials for the expression of genetic information. The concurrence of BBR interacting with DNA double strands and mRNA poly (A) tails with a “thymidine and adenosine” preference directed us to explore the details of the mechanism by which BBR regulates gene expression^12^. To decipher the role of BBR’s nucleotide-binding ability in gene regulation, we used a number of genes with or without TATA boxes as models for mechanistic studies. In the wild type cells, BBR down-regulated the mRNA expression of genes containing TATA boxes in their promoter regions, including *Hsp70*, *Il-6*, *Il-1β*, *C-fos*, *Pparγ* and *Tnfα*^13^,^14^,^15^,^16^,^17^ (Fig. 2a–f). Conversely, BBR treatment increased the mRNA levels of genes that lack TATA boxes, including *RB1*, *Kras*, *p53*, *Sp1*, *Pi3k* and *Akt*^18^,^19^,^20^,^21^,^22^,^23^ (Fig. 2g–l). These results indicated a role for BBR binding to TATA boxes in gene promoters in the inhibition of gene transcription and indirectly suggested that BBR might also bind to poly (A) tails and consequently increase mRNA levels. When the gene transcription was inhibited by Actinomycin D, BBR protected RNA from degradation, irrespective of the presence of a TATA box (Supplementary Fig. S2). When the poly (A) tail was removed, accelerated mRNA degradation was observed, suggesting that the poly (A) tail might indeed be the target through which BBR protects mRNA from degradation (Supplementary Fig. S3).

### Recombination of HSP70 and Rb gene promotor to verify BBR effects

To further clarify how BBR interacts with TATA boxes and poly (A) tails, we chose as examples the HSP70 gene, which contains a TATA box^24^, and the Rb gene, which lacks a TATA box^25^. *Hsp70* has a TATA box in its transcriptional regulatory region, and it responds to heat stress. The expression of this gene was suppressed by BBR *in vivo* and *in vitro* (Supplementary Fig. S4). *RB1* (the gene encoding Rb) is a TATA box-independent but GC box-dependent gene, and it responds to ischemia-reperfusion stress. *RB1* was up-regulated by BBR *in vivo* and *in vitro*, as in our previous report^12^.

**Figure 3:**
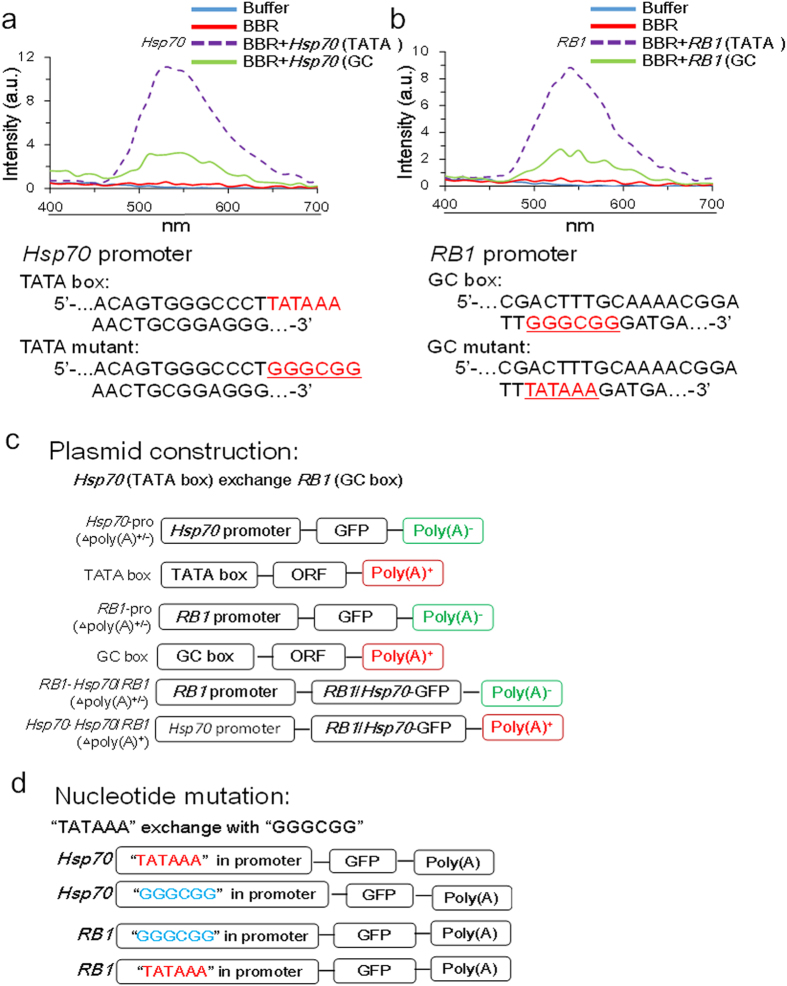
Plasmid construction for TATA box – GC box exchange. (**a**): Fluorescence spectrum of *Hsp70* with a TATA box compared with that of an *Hsp70* mutant containing a “GGGCGG” sequence. BBR could clearly bind to the TATA box, increasing fluorescence, but BBR failed to increase fluorescence when the TATA sequence was replaced with GC pairs, indicating that BBR did not bind to the GC box. (**b**): Fluorescence spectrum of *RB1* with a GC box compared with that of an *RB1* mutant containing the sequence “TATAAA”. (**c**): Schematic of *Hsp70* and *RB1* promoter exchange plasmid construction. (**d**): Schematic of “TATAAA” and “GGGCGG” mutant plasmid construction.

Using recombinant DNA technology, engineered plasmids containing a TATA box or a GC box were constructed (Fig. 3C; Supplementary Fig. S5). Plasmids with or without poly (A) tails were also applied to confirm the effects of BBR on poly (A) tails. The GFP gene was added to all of the plasmids to illuminate BBR’s effects without disturbing endogenous genes (Supplementary Fig. S6)^26^.

**Figure 4:**
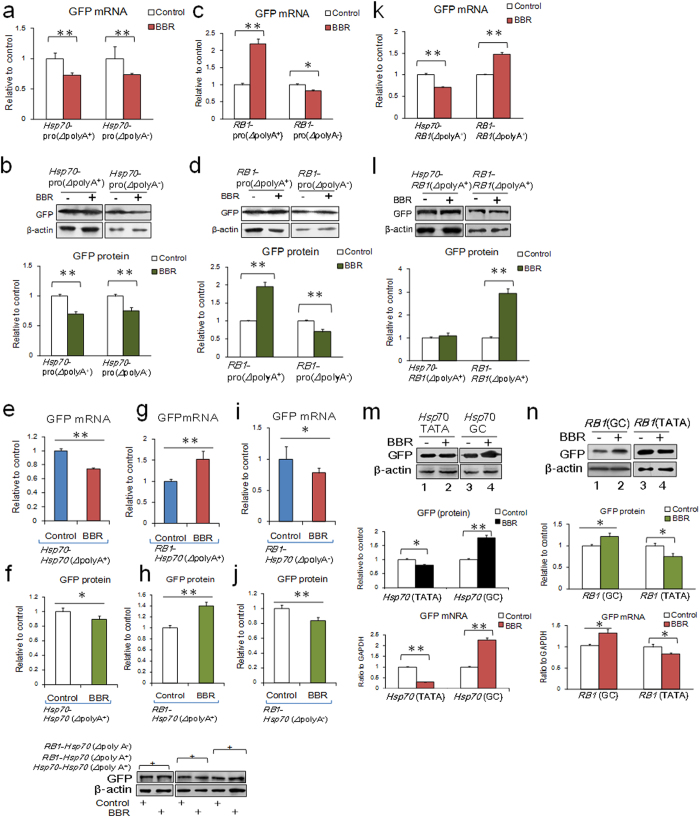
Effect of berberine on mRNA and protein expression levels with the TATA box and GC box of *Hsp70* and *RB1*. (**a,b**): mRNA and protein expression of GFP with or without poly (A) tails transcribed by the *Hsp70* promoter. (**c,d**): mRNA and protein expression levels of GFP with or without poly (A) tails transcribed by the *RB1* promoter. (**e,f**): mRNA and protein expression of an *Hsp70*-GFP fusion protein containing the *Hsp70* promoter. (**g,h**): mRNA and protein expression of an *Hsp70*-GFP fusion protein containing the *RB1* promoter and a poly (A) tail. (**i,j**): mRNA and protein expression of an *Hsp70*-GFP fusion protein containing the *RB1* promoter without a poly (A) tail. (**k,l**): mRNA and protein expression levels of a *RB1*-GFP fusion protein containing the *Hsp70* or *RB1* promoter. (**m,n**): mRNA and protein expression levels of a *Hsp70*-GFP or *RB1*-GFP fusion protein containing the “TATA”/“GC” sequence in the promoter. The data are expressed as the mean ± S.D. from six independent experiments. *, **P < 0.05, *P* < 0.01 *vs.* control groups. Full-length gels and blots are included in Supplementary Figure 7.

*Hsp70* contains both a TATA box in its promoter region and a poly (A) tail on its mRNA. The down-regulation of *Hsp70* mRNA levels observed upon BBR treatment indicates that the inhibitory effect of BBR on the TATA box dominates in the presence of both a TATA box and a poly (A) tail (Fig. 4a,b). The binding affinity of BBR to the *Hsp70* promoter declined when the TATA box was replaced by a GC-rich domain (Fig. 3a,b). Because of the limited distribution of BBR in cells and its lower affinity for GC-rich sequences^9^, it might be difficult for BBR to achieve an effective dosage to affect GC box-dependent gene transcription. In this case, we hypothesize that the activity of BBR on poly (A) tails becomes the principal effect in the absence of a TATA box, resulting in enhanced mRNA stability and up-regulation at the protein level. *RB1* contains both a GC box in its transcriptional initiation region and a poly (A) tail on its mRNA. The level of *RB1* mRNA increased following BBR treatment, attributable to lower mRNA degradation^12^. The BBR-dependent *RB1* mRNA up-regulation was abolished with poly (A) depletion (Fig. 4c,d). These data collectively indicate that the TATA box is the principal target of BBR for transcriptional inhibition *in vivo*. However, for TATA box-independent genes, BBR can shift its primary target to the poly (A) tails of mRNAs, leading to up-regulation of protein levels through enhancement of mRNA stability.

To further confirm the specificity of the TATA box target, gene cross-recombination experiments were conducted. We recombined the *Hsp70* open reading frame (ORF) with the *RB1* gene promoter region (containing a GC box) and simultaneously compared the effects of BBR on synthesized RNA fragments with or without a poly (A) tail. When the ORF was driven by the *Hsp70* promoter (containing a TATA box), BBR treatment inhibited its expression. Conversely, when the *Hsp70* promoter was replaced with the *RB1* promoter, the inhibitory effect of BBR on *Hsp70* expression disappeared, and, in turn, the expression of *Hsp70* mRNA increased due to BBR’s effect on the poly (A) tail. When the poly (A) tail of *Hsp70* (driven by the GC box-containing *RB1* promoter) was removed, the increase in *Hsp70* expression was abolished (Fig. 4e–j). In the opposite experiment, when the promoter region of the *RB1* gene was replaced with the *Hsp70* promoter region containing the TATA box, the effect of BBR on *RB1* expression switched from up-regulation to inhibition because of BBR’s TATA box-mediated inhibitory effect (Fig. 4k,l).

In general, the sequence of a TATA box consists of “TATAAA” in the gene start transcription region and the sequence of a GC box consists of “GGGCGG” in the gene start transcription region. When the nucleotide sequence “TATAAA”, which is the TATA box, in the *Hsp70* gene promoter region was replaced with “GGGCGG”, which is the GC box, the inhibitory effect of BBR on *Hsp70* gene transcription disappeared. When the “GGGCGG” sequence in the *RB1* gene promoter region was replaced with “TATAAA”, BBR acquired an inhibitory effect on *RB1* gene transcription (Fig. 3d). These results demonstrate that the “TATAAA” nucleotide sequence is a functional target of BBR in its inhibition of gene transcription (Fig. 4m,n). Because of TATA box in gene transcription start site or the absence, BBR would exhibit a variety of effects.

### Amplified effects of BBR on the nucleotides in pathophysiological conditions

**Figure 5:**
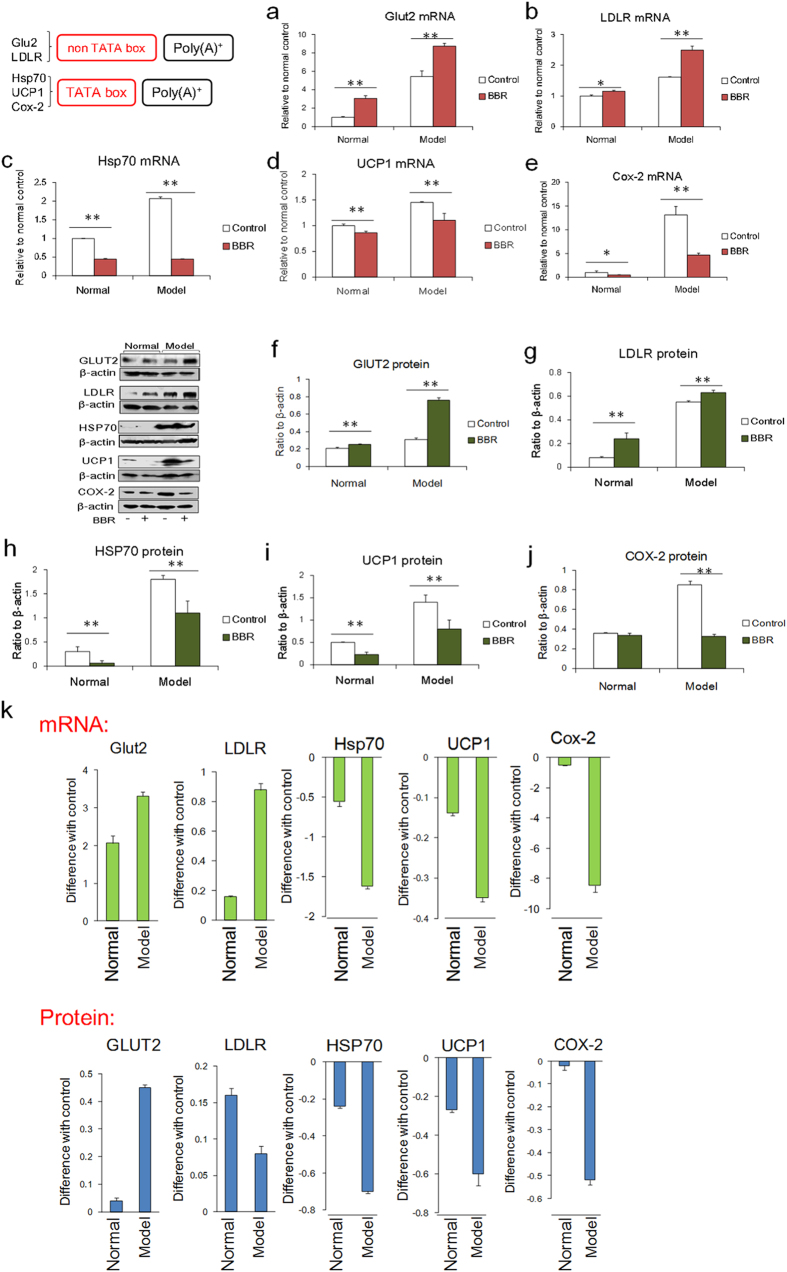
Expression of cytokines with or without TATA boxes in wild type cells under both physiological and pathophysiological conditions. HepG2 cells were used for *GLUT2* and *LDLR*, which lack TATA boxes, while PC12 cells were used for *Hsp70* and *Cox-2*, and HIB cells were used for *Ucp1*, which contain TATA boxes. (**a,b**): mRNA expression levels of *GLUT2* and *LDLR*. (**c–e**): mRNA expression of *Hsp70*, *Ucp1* and *Cox-2*. (**f,g**): Protein expression levels of GLUT2 and LDLR. (**h–j**): Protein expression of HSP70, UCP1 and COX-2. (**k**): Differences between normal and model conditions. mRNA expression levels are displayed in green, and protein expression is shown in blue. BBR had stronger effects under model conditions relative to normal conditions. BBR was applied at 0.8 μg/ml. The data are expressed as the mean ± S.D. from six independent experiments. *, **P < 0.05, *P* < 0.01 *vs.* control groups. Full-length gels and blots are included in Supplementary Figure 8.

Due to the strong affinity between BBR and nucleotides, BBR might directly act to loosen packed nucleotides as soon as it enters the cell^9^. Furthermore, the gene structure-dependent regulatory activities of BBR might be the fundamental mechanism underlying its diverse pharmacological activities. In HepG2 cells, both *GLUT2* and *LDLR* are GC box-dependent and poly (A) tail-containing genes^27^,^28^. BBR did not significantly influence their baseline mRNA levels under normal conditions. However, when cells suffer sugar and lipid overload, the transcriptional regions of these genes are loosened, and their mRNA levels are stimulated. BBR application significantly further elevated their expression levels under these conditions. It is likely that BBR could not effectively inhibit the GC box-independent gene transcription but could prolong mRNA half-life through binding to the mRNA poly (A) tail (Fig. 5a,b), which subsequently resulted in up-regulated protein production (Fig. 5f,g). Conversely, BBR suppressed the baseline mRNA levels of TATA box-dependent and poly (A) tail-containing genes such as *Hsp70*, *Ucp1*, and *Cox-2*^29^,^30^ under normal conditions. The application of pathophysiological models further opened the transcriptional control region of these genes and increased their expression in response to stress. Under these conditions, BBR administration further strongly inhibited the expression of these genes (Fig. 5c–e), as the TATA box-dependent inhibitory effect of BBR is its primary activity compared with its effect on mRNA. The protein production levels in response to BBR were consistent with the changes in mRNA levels (Fig. 5h–j), indicating that the translational component of gene expression might not be affected by BBR.

After entering into cells, BBR could directly bind to a TATA box or a poly (A) tail. When BBR interacts with TATA boxes in gene promoters, it might hinder the reorganization of TATA-binding protein (TBP) on the TATA box, inhibiting the initiation of gene transcription. When BBR binds to poly (A) tails, it may enhance the stability of the mRNA, thus leading to a relatively higher protein level. Under pathophysiological conditions, the more loosely packed transcriptional regulatory regions allow easier access of BBR to DNA, which could lead to enhanced pharmacological activities of BBR based on its inhibitory effect on the transcription of TATA box-dependent genes. As for GC box-dependent genes, because BBR cannot reach an effective dosage to influence the transcription of these genes in living cells, the interaction between BBR and the poly (A) tail could prolong the half-life of the mRNA and subsequently maintain relatively higher protein levels (Fig. 5k), which are important for BBR’s pharmacological activity in some diseases. As an intercalator, BBR’s effects manifest as inhibited or increased gene transcription and protein production for a large number of genes. This situation occurs in pathological conditions such as venereal disease stress, indicating differences in the effects of BBR in physiological and pathological conditions.

### Definition of “drug target code” of BBR

In addition to the TATA box, GC box, and poly (A) tail, other gene expression-related elements composed of variant nucleotide sequences could also be targets of BBR in biological systems. Due to the limited cell-compartment distribution of BBR and the different binding affinities of BBR to adenine, cytosine, guanine, and thymine, BBR has a unique activity toward each element that is determined by the individual nucleotide components. We propose that this phenomenon underlies the concept of a “drug target code”. This code is defined as the complex arrangement of drug-interacting components of a functional drug target, with the final drug activity emerging from the accumulated outcomes of all of the drug-component interactions.

## Conclusion

In summary, this is the first study to comprehensively explore how BBR regulates gene expression through simultaneous direct interactions with both DNA and mRNA. BBR can interact with the TATA box transcriptional element to inhibit gene transcription and concurrently interact with mRNA in a poly (A)-dependent manner to prolong mRNA half-life, which subsequently results in relatively higher gene expression levels. The outcome of the BBR-regulated gene expression profile thus depends on the structure of the individual genes. We conclude that thymidine and adenosine are the actual targets of BBR in biological systems. Different “T/A” compositions in individual genes may function as codes that determine their accessibility to BBR, resulting in different responses to drug treatment. This evidence-supported hypothesis provides a novel understanding of the interactions between chemical compounds and biological targets, explaining the multiple activities of a single compound (such as BBR) in different physiological contexts.

## Methods

### Animals

The ICR mice (male, 6–8 weeks old, 20–22 g) used in this study were purchased from Vital River Laboratories (Beijing, China) and kept in an SPF (specific pathogen free) room (temperature: 25 ± 1 °C, air humidity: 50% ± 10%). Mice were fed with sterile water and the standard laboratory chow diet *ad libitum*. The laboratory animal facility has been accredited by AAALAC (Association for Assessment and Accreditation of Laboratory Animal Care International). All experimental procedures were approved by the IACUC (Institutional Animal Care and Use Committee) of Tsinghua University and carried out in accordance with the People’s Republic of China Legislation Regarding the Use and Care of Laboratory Animals (Approval ID: 2014-DuLJ-BBRDNRRNA).

### Fluorescence measurement

The fluorescence analysis was performed in a black microplate (384-well, Corning, USA) on a Multifunctional Microplate Reader (Varioskan Flash, Thermo Scientific, USA). The excitation wavelength was 350 nm. All of the reactions were carried out in 4 mM BPES buffer (pH = 7) at room temperature, as described previously^31^. Blank buffer, RNA, DNA and BBR alone were also measured as background and controls. Double-stranded DNA oligos were obtained from mix of complementary oligos (equal molar concentrations) by denaturing at 95 °C for 5 min and then slow cooling to room temperature over a period of 1 h. The DNA oligonucleotides were synthesized by Sangon Biotech (Shanghai, China) and the RNA oligos with poly (A) tails by Sigma Aldrich (USA). The sequences of all DNA and RNA oligos used in these experiments are shown in Supplementary Tables 1 & 2.

### Heat stress model *in vivo*

In the *in vivo* study, the mice were randomly divided into 4 groups, including two normal groups and two hot model groups. The two normal groups were kept at room temperature; one was treated with normal saline and served as a control, and the other was treated with BBR (0.8 mg/kg) by intravenous administration. The two hot model groups were first injected with normal saline or BBR (0.8 mg/kg); 1 h after injection, the mice were exposed to 40 °C in a constant thermal TS-1 Incubator (Huangshi Medical Instrument, China) for 2 h^32^. After the end of the heat stress, the brains of the mice were immediately isolated and kept at −80 °C for RNA and protein tests.

### Oxygen-glucose deprivation (OGD) *in vitro*

The model of OGD followed by reperfusion was used to mimic cerebral ischemia *in vitro*^33^. PC12 cells were seeded into 6-well plates and maintained in RPMI-1640 containing 10% HS and 5% FBS under 5% CO_2_ at 37 °C. BBR (0.8 μg/ml) was pre-treated for 15 h prior to OGD in the drug group. In the model group, the culture medium was changed to Earle’s solution without glucose, and the cell plate was placed in a humidified atmosphere without oxygen (95% N_2_ and 5% CO_2_) at 37 °C for 2 h. The medium was then replaced with normal medium, and the cells were maintained under normal conditions for another 6 h as reperfusion.

### *In vitro* models of heat and cold stress

The high temperature model was used to study the cells’ heat shock responses to high temperature stress *in vitro*, and the low temperature model was used to test responses to cold stress. PC12 cells, used for the high temperature model. And HIB cells, used for the low temperature model, were seeded into 6-well plates. BBR (0.8 μg/ml) was pre-treated for 15 h in the drug group. In the model groups, cells were exposed to a high temperature of 43 °C for 2 h or a low temperature of 4 °C for 1 h^34^.

### *In vitro* models of glucose and lipid overload

The glucose and lipid overload models were, respectively used to mimic the pathological conditions of hyperglycemia and hyperlipidemia *in vitro*. HepG2 cells were maintained in DMEM containing 10% FBS. Cells were seeded into 6-well plates. BBR (0.8 μg/ml) was pre-treated for 2 h in the drug group. In the glucose overload model, cells were treated with 50 mg/ml glucose diluted in normal medium for 24 h. In the lipid overload model, cells were treated with 20 μg/ml Triton WR 1339 for 24 h.

### Quantitative PCR

The quantitative PCR (qPCR) protocol followed that of Jiang *et al.*^32^ All primer sequences used in these analyses are shown in Supplementary Table 3^35^,^36^,^37^,^38^,^39^,^40^,^41^,^42^,^43^,^44^,^45^,^46^,^47^,^48^,^49^.

### Western blot analysis

Western blot was performed following a previously described protocol^4^. β-actin served as an internal control. The primary antibodies for GLUT2, COX2, and UCP1 were purchased from Bioworld (USA). Primary antibodies for HSP70, LDLR, GFP and β-actin were purchased from Abcam (UK), Proteintech (USA), Biosmart (USA) and Santa Cruz (USA), respectively. Secondary antibodies conjugated to horseradish peroxidase (HRP) were also purchased from Santa Cruz (USA).

### Construction of pEGFP-N1 (ΔpolyA^-^)

The plasmid pEGFP-N1 was provided by Professor Ye-Guang Chen (Tsinghua University, China). The 1550–1590 poly (A) signal was deleted by amplifying nucleotides 1–1550 as Fragment 1 and nucleotides 1590–4730 as Fragment 2, double-digesting using AseI and EcoRV, and then ligating into a circular plasmid, termed pEGFP-N1 (ΔpolyA^−^). The primer sequences were designed using Primer Premier 5.0 software as follows: Fragment 1, sense: 5′-TAGTTATTAATAGTAATCAATTACGG-3′ (AseI), antisense: 5′-ACCGATATCAGCTGCAATAAACAAGTT-3′(EcoRV); Fragment 2, sense: 5′-CATGATATCCACTGCATTCTAGTTGTG-3′ (EcoRV), antisense: 5′-GGCTATGAACTAATGACCCCGTAAT-3′. The AseI restriction site was in the PCR product, not the primer.

### Construction of Hsp70/RB1-pro-Hsp70/RB1 (ΔpolyA^+/−^) plasmids

The *Hsp70* and *RB1* promoters were cloned by PCR from the genome of PC12 cells, extracted with a Genomic DNA Kit (Tiangen Biotech., China). The plasmids *Hsp70*-pro(ΔpolyA^+^) and *Hsp70*-pro(ΔpolyA^−^) were constructed by replacing the CMV promoters of pEGFP-N1 and pEGFP-N1 (ΔpolyA^−^) with the *Hsp70* promoter, using the restriction endonucleases AseI and EcoRI. The *Hsp70* and *RB1* ORFs were cloned from PC12 cDNA obtained by RT-PCR. The *Hsp70* and *RB1* ORFs were inserted into *Hsp70*-pro(ΔpolyA^+^) using BamHI to generate the *Hsp70*-pro-*Hsp70*(ΔpolyA^+^) and *Hsp70*-pro-Rb(ΔpolyA^+^) plasmids. *RB1*-pro(ΔpolyA^+^), *RB1*-pro(ΔpolyA^−^), *RB1*-pro-*Hsp70*(ΔpolyA^+^), *RB1*-pro-Rb(ΔpolyA^+^) and *RB1*-pro-*Hsp70*(ΔpolyA^−^) were constructed in a similar way. Cloning primers were as follows: *Hsp70* promoter sense: 5′-TGGGAGAGGAGAGTGTGTCG-3′, antisense: 5′- GGGCGGAGAAGATCTCGAAG-3′; *RB1* promoter sense: 5′-TCTTTGTAGCTGGACCTGGGCCT-3′; antisense: 5′-GGGAGCCAGCGAGCTGTGGAG-3′; *Hsp70* ORF sense: 5′-ATGTCGGTGGTGGGCATAGAC-3′; antisense: 5′-ATCAATGTCCATCTCAGGAAGC-3′; *RB1* ORF sense: 5′-ATGCCGCCCAAAACCCCCCGAAAAACGGCC-3′; antisense: 5′-AGCATGGATACCTCAAACAAGGAAGAGAAA-3′^12^. 293T cells were transfected with all types of plasmids and sorted using a BD AriaIII (Becton Dickinson, USA).

### Construction of Hsp70-GC-pro and RB1-TA-pro mutant plasmids

The TATA box (TATAAA) in the *Hsp70* promoter was altered to a GC box (GGGCGG), and the GC box in the *RB1* promoter was changed to a TATA box using overlap extension-PCR. The overlap site-direct mutagenesis was performed as previously described^50^. In addition to the cloning primers for the *Hsp70* and *RB1* promoters, the primers used for this experiment were as follows: *Hsp70* site-direct mutant primer sense: 5′-ACAGTGGGCCCTGGGCGGAACTGCGGAGGGCTT-3′, antisense: 5′-AAGCCCTCCGCAGTTCCGCCCAGGGCCCACTGT-3′; *RB1* site-direct mutant primer sense: 5′-GACGTGACGCCGCTATAAAAAGTGACGTTTTCCC-3′, antisense: 5′-GGGAAAACGTCACTTTTTATAGCGGCGTCACGTC-3′. The plasmids were also transfected into 293T cells and sorted by flow cytometry.

### Construction of the SP64-GFP plasmid and *in vitro* transcription

The pSP64 polyA vector was purchased from Promega (USA). The GFP ORF was cloned from the vector pEGFP-N1 and inserted into the pSP64 polyA vector by HindIII to obtain the SP64-GFP plasmid. The primers used to clone GFP were as follows: antisense: 5′-GATCCACCGGTCGCCACCA-3′; sense: 5′-GTACAGCTCGTCCATGCCGAGAGT-3′. The plasmid SP64-GFP was separately digested with SacI or EcoRI to generate two different types of linear transcriptional templates. Two types of GFP RNA, without or with a 30-nt poly (A) tail, were obtained in a cell-free *in vitro* transcription system containing NTP mix, RNase inhibitor (Transgene, China), and SP64 RNA polymerase (NEB, USA). DNA templates were digested by RNase-free DNase, and then RNA transcripts were purified using phenol chloroform.

### Preparation of the S20 fraction from cells and RNA degradation *in vitro*

PC12 cells were harvested by centrifugation at 500 g for 5 min at 4 °C and washed once with ice-cold PBS. The cell pellet was suspended in ice-cold buffer A (~200 μl per 1 × 10^7^ cells) with protease inhibitor cocktail and disrupted by beating 15 times with a sterile pestle. The nuclei were centrifuged at 2500 g for 10 min at 4 °C. The supernatant was further centrifuged at 13000 g for 30 min at 4 °C to remove cell debris. The resulting supernatant, named the S20 fraction, was stored at −80 °C^51^.

pEGFP-N1 and pEGFP-N1 (ΔpolyA^-^) were transfected and expressed in 293T cells. RNA was extracted using the RNA pre-pure Cell/Bacteria Kit (Tiangen, China) to obtain GFP mRNA with or without a poly (A) tail. The system of RNA degradation *in vitro* was developed as follows: 0.13 μl BBR (50 μg/ml) (substituted with water in the control group; both added before reverse transcription), 0.83 μl 100 mM ATP, 1.25 μl RNA, and 4.17 μl of the PC12 S20 fraction^52^. The RNA degradation mixture was incubated at 37 °C for different durations (0, 15, 30 min), then terminated by adding RNase Inhibitor and placed on ice immediately. The mix was reverse-transcribed using random primers and EasyScript Reverse Transcriptase enzyme (Transgene, China). Quantitative PCR was carried out to detect mRNA decay. The degradation of GFP RNA with or without a poly (A) tail generated by *in vitro* transcription was also detected by this method.

### NMR spectroscopy for the binding between BBR and AMP

AMP2Na (purity ≥99%) was purchased from Xiasi Biotech (Beijing, China). The NMR analyses of AMP2Na, berberine and the mixture of the two were carried out in the Analysis Center of Tsinghua University. The HMBC (Heteronuclear Multiple-Bond Correlation) spectra were obtained at 600 MHz (^1^H) and 150 MHz (^13^C) on a JNM-ECA600 spectrometer in D_2_O at 23.6 °C^53^.

### Data analysis

All data were expressed as the mean ± S.D. Student’s *t*-tests for statistical analysis was performed using Excel Software for Office 2013 (Microsoft, U.S.). Image processing for quantitative data of protein expression was performed using Quality ONE4.62 (BIO-RAD, U.S.). *P* < 0.05 was considered statistically significant.

## Additional Information

**How to cite this article**: Yuan, Z.-Y. *et al.* TATA boxes in gene transcription and poly (A) tails in mRNA stability: New perspective on the effects of berberine. *Sci. Rep.* **5**, 18326; doi: 10.1038/srep18326 (2015).

## Supplementary Material

Supplementary Information
