# Supplementary material for: TATA boxes in gene transcription and poly (A) tails in mRNA stability: New perspective on the effects of berberine
Source: Sci Rep. 2015 Dec 16;5:18326. doi: 10.1038/srep18326 (PMC4680869; doi:10.1038/srep18326)
Supplement: Supplementary Information [file srep18326-s1.doc]

**Supplementary Figures**

**TATA boxes in gene transcription and poly (A) tails in mRNA stability: New perspective on the effects of berberine**

Zhi-Yi Yuan^1^†, Xi Lu^1^†, Fan Lei^1^†, Yu-Shuang Chai^1^†, Yu-Gang Wang^2^, Jing-Fei Jiang^1^,

Tian-Shi Feng^1^, Xin-Pei Wang^1^, Xuan Yu^1^, Xiao-Jin Yan^1^, Dong-Ming Xing^1^, Li-Jun Du^1*^

^1^MOE Key Laboratory of Protein Sciences, Laboratory of Molecular Pharmacology and Pharmaceutical Sciences, School of Life Sciences and School of Medicine, Tsinghua University, Beijing 100084, China

**Supplementary Figure 1.**

**Supplementary Figure 1. NMR analysis of BBR binding to AMP^2-^.** To further study the binding sites of BBR and poly (A), a detailed 2D NMR for ^1^H-^13^C cosy (gradient-enhanced HMBC) was carried out for the binding between AMP^2-^ (monomer of poly (A)) and BBR. (a): Chemical structure of AMP^2-^. (b): Chemical structure of BBR. (c): Overlay of the HMBC spectra of 30 mM BBR in the absence (red) and presence (green) of 60 mM AMP^2-^. (d): Selected parts of the BBR NMR spectrum in the absence and presence of AMP^2-^. Chemical shift changes are indicated by the arrows. With or without AMP, the spots corresponding to the protons 8- and 5-H were coincident. However, several chemical shift changes were observed. The spots corresponding to 13-, 12-, 1- and 4-H moved to the high filed, and the spot corresponding to 15-H moved in the opposite direction, to the low filed (d). These results suggested that there was an electrophilic group in AMP^2-^ acting on the 1, 4, 12, 13-H of BBR as well as a strong electronegative group in AMP^2-^ acting on 15-H of BBR. When NMR spectra of AMP were analyzed, there were no significant chemical shift differences observed with or without BBR (data not shown). This implied that the BBR’ binding site on AMP^2-^ may be nitrogen or oxygen. The electrophilic group may be -NH_2_, whereas the strong electronegative group is more likely to be the phosphate group.

**Supplementary Figure 2.**

**Supplementary Figure 2. Effect of berberine on mRNA degradation in PC12 cells**. BBR (0.8 μg/ml) was pre-treated for 15 h in the drug group. Next, in both the control and BBR groups, actinomycin D (5 μg/ml) was administered for different durations (0, 15, 30, 60 min). The mRNA abundances of several genes were detected by qPCR. The data are expressed as the mean ± S.D. from three independent experiments.

**Supplementary Figure 3.**

**Supplementary Figure 3. Effect of berberine on GFP mRNA with or without poly (A) tail degradation by the S20 fraction in a cell-free system.** (a-c): mRNA extracted from 293T cells transfected with the pEGFP-N1 or pEGFP-N1 (*Δ*polyA^-^) plasmid. (b): GFP mRNA with a poly (A) tail. (c): GFP mRNA without a poly (A) tail. (d-f): RNA transcribed in vitro using the linearized SP64-GFP plasmid as a template. (e): GFP mRNA with a poly (A) tail. (f): GFP mRNA without a poly (A) tail. (a, d): RT-PCR with random and Oligo(dT) primers to effectively identify the presence or absence of the poly (A) tail. The data are expressed as the mean ± S.D. from three independent experiments. ** *P* < 0.01 *vs.* control groups.

**Supplementary Figure 4.**

**Supplementary Figure 4. Effect of berberine on HSP70 expression under normal and heat stress conditions in mice and PC12 cells**. (a): *Hsp70* mRNA expression in the mouse brain. (b): HSP70 protein expression in the mouse brain. (c): *Hsp70* mRNA expression in PC12 cells. (d): HSP70 protein expression in PC12 cells. Heat was applied at 40 °C for 2 h in the *in vivo* experiment, whereas PC12 cells were stressed at 40 °C for 2 h. The dose of BBR was at 0.8 mg/kg by intravenous injection. The data are expressed as the mean ± S.D. from six independent mice. ## *P* < 0.01 *vs.* normal control groups; *, ** *P* < 0.05, *P* < 0.01 *vs.* the model control groups.

**Supplementary Figure 5.**

**Supplementary Figure 5.** Recombination of Hsp70 and RB1 promoter plasmids.

**Supplementary Figure 6.**

**Supplementary Figure 6. Protein expression of GFP in 293T cells.** In the control groups, the expression of GFP couldn’t be detected because there is no GFP gene in the mammalian cells (lane 1). When the plasmids of GFP mRNA driven by Hsp70 and RB1 promoter was transfected to the cells, the expression of GFP could be observed (lane 2 and 3).

**Supplementary Figure 7.**

**Supplementary Figure 7. Effect of berberine on mRNA and protein expression levels with the TATA box and GC box of *Hsp70* and *RB1*.** (b): protein expression of GFP with or without poly (A) tails transcribed by the *Hsp70* promoter. (d): protein expression levels of GFP with or without poly (A) tails transcribed by the *RB1* promoter. (f): protein expression of an *Hsp70*-GFP fusion protein containing the *Hsp70* promoter. (h): protein expression of an *Hsp70*-GFP fusion protein containing the *RB1* promoter and a poly (A) tail. (j): protein expression of an *Hsp70*-GFP fusion protein containing the *RB1* promoter without a poly (A) tail. (l): protein expression levels of a *RB1*-GFP fusion protein containing the *Hsp70* or *RB1* promoter. (m - n): protein expression levels of a *Hsp70*-GFP or *RB1*-GFP fusion protein containing the “TATA” / “GC” sequence in the promoter. All blots were run under the same conditions and analyzed in a similar way.

**Supplementary Figure 8.**

**Supplementary Figure 8. Expression of cytokines with or without TATA boxes in wild type cells under both physiological and pathophysiological conditions.** HepG2 cells were used for *GLUT2* and *LDLR*, which lack TATA boxes, while PC12 cells were used for *Hsp70* and *Cox-2*, and HIB cells were used for *Ucp1*, which contain TATA boxes. BBR was applied at 0.8 μg/ml. All blots were run under the same conditions and analyzed in a similar way.
